# Heterogeneidade regional no cumprimento das diretrizes do Programa Nacional de Alimentação Escolar: uma análise comparativa entre Nordeste e Sul do Brasil com aprendizado de máquina

**DOI:** 10.1590/0102-311XPT056125

**Published:** 2026-03-23

**Authors:** Bruno Herrera Sgura, Maria Sylvia Macchione Saes, Camila Veneo Campos Fonseca, Yasmin Proença Bueno Adorno

**Affiliations:** 1 Universidade de São Paulo, São Paulo, Brasil.; 2 Universidade Estadual de Campinas, Campinas, Brasil.

**Keywords:** Política Pública, Alimentação Escolar, Aprendizado de Máquina, Agricultura, Public Policy, School Feeding, Machine Learning, Agriculture, Política Pública, Alimentación Escolar, Aprendizaje Automático, Agricultura

## Abstract

O Programa Nacional de Alimentação Escolar (PNAE) estabelece a obrigatoriedade de que ao menos 30% dos recursos federais transferidos às entidades executoras sejam destinados à aquisição de alimentos da agricultura familiar. No entanto, os resultados alcançados com o programa apresentam elevada heterogeneidade regional. Este estudo utiliza a abordagem de aprendizado de máquina, particularmente árvores de classificação, para identificar os fatores que mais influenciam o cumprimento da legislação do PNAE em duas regiões brasileiras, Nordeste e Sul, a partir de dados de 2.650 municípios, referentes ao ano de 2017. A análise dessas regiões justifica-se pela elevada presença da agricultura familiar, ainda que com diferenças significativas no cumprimento da meta dos 30%: no Sul, 77% dos municípios cumprem a regra, enquanto no Nordeste, a adesão é de 38%. Os resultados da pesquisa indicam que as diferenças entre as regiões estão associadas a fatores como: limitações financeiras dos municípios e menor formalização dos estabelecimentos agrícolas. Além disso, aspectos como o tamanho do município, o grau de centralização na aquisição de alimentos e o perfil da produção agrícola local também influenciam o cumprimento da legislação. Conclui-se que políticas públicas devem considerar especificidades regionais para melhorar a adesão e a eficácia da implementação no âmbito regional.

## Abstract (en)

The Brazilian National School Feeding Program (PNAE, acronym in Portuguese) establishes the obligation that at least 30% of federal resources transferred to the executing boards be allocated to the acquisition of food from family farms. However, the results achieved with the program reveal high regional heterogeneity. This study used the machine learning approach (particularly classification trees) to identify factors that most influence compliance with PNAE legislation in the Northeast and South regions of Brazil based on data from 2,650 municipalities referring to the year 2017. The analysis of these two regions is justified by the high presence of family farming, although with significant differences in the fulfillment of the 30% target: 77% of municipalities in the South meet this target, compared to 38% in the Northeast region. The results of the survey indicate that differences between the two regions are associated with factors such as financial limitations of the municipalities and less formalization of agricultural establishments. Moreover, aspects such as the size of the municipality, degree of centralization in the acquisition of food, and profile of local agricultural production also influence compliance with legislation. Therefore, public policies should consider regional specificities to improve adherence and the effectiveness of implementation on the regional level.

## Abstract (es)

El Programa Nacional de Alimentación Escolar (PNAE) establece la obligación de que al menos el 30 % de los recursos federales transferidos a las entidades ejecutoras se destinen a la adquisición de alimentos procedentes de la agricultura familiar. Sin embargo, los resultados obtenidos con el programa presentan una gran heterogeneidad regional. Este estudio utiliza el enfoque del aprendizaje automático, en particular los árboles de clasificación, para identificar los factores que más influyen en el cumplimiento de la legislación del PNAE en dos regiones brasileñas, el noreste y el sur, a partir de datos de 2.650 municipios correspondientes al año 2017. El análisis de estas regiones se justifica por la elevada presencia de la agricultura familiar, aunque con diferencias significativas en el cumplimiento del objetivo del 30%: en el sur, el 77% de los municipios cumplen la norma; mientras que, en el noreste, la adhesión es del 38%. Los resultados de la investigación indican que las diferencias entre las regiones están asociadas a factores como: las limitaciones financieras de los municipios y la menor formalización de los establecimientos agrícolas. Además, aspectos como el tamaño del municipio, el grado de centralización en la adquisición de alimentos y el perfil de la producción agrícola local también influyen en el cumplimiento de la legislación. Se concluye que las políticas públicas deben tener en cuenta las especificidades regionales para mejorar la adhesión y la eficacia de la implementación a nivel regional.

## Introdução

O Programa Nacional de Alimentação Escolar (PNAE) é uma das políticas públicas mais relevantes para a segurança alimentar e nutricional no Brasil e no mundo. Ele garante, de forma universal e gratuita, o acesso a refeições compostas por alimentos diversificados, frescos e minimamente processados a todos os alunos da rede pública nacional ^1^.

As políticas de alimentação escolar no Brasil remontam à Campanha Nacional pela Alimentação da Criança, de 1935, passando por reformulações até a criação do PNAE em 1979 ^2^ ^,^ ^3^. A descentralização do programa ganhou força com a Constituição de 1988, que garantiu o direito à alimentação escolar e delegou responsabilidades a estados e municípios. A *Lei nº 11.947*/2009 ^4^, por sua vez, consolidou a integração entre o PNAE e a agricultura familiar, determinando que pelo menos 30% dos recursos repassados pelo Fundo Nacional de Desenvolvimento da Educação (FNDE) fossem destinados à compra de alimentos de produtores familiares.

Essa legislação fortaleceu a agricultura familiar, associando o PNAE à promoção da economia local e ao desenvolvimento rural. Além de priorizar comunidades indígenas, quilombolas e assentamentos da reforma agrária, buscou reduzir as disparidades regionais e aumentar a eficiência do programa. A vinculação do PNAE à agricultura familiar também impulsionou os circuitos locais de abastecimento, elevando a renda de produtores e combatendo a insegurança alimentar nas comunidades atendidas ^5^.

A partir da descentralização, parte importante da literatura avaliou os desafios no cumprimento da meta de compra da agricultura familiar ^6^ ^,^ ^7^, indicando elevada heterogeneidade nos resultados alcançados com a implementação do PNAE a nível local ^8^ ^,^ ^9^ ^,^ ^10^. No entanto, a maioria dos estudos se concentra em estudos de caso ^11^ ^,^ ^12^ ^,^ ^13^ ^,^ ^14^ ^,^ ^15^ ^,^ ^16^ e análises de determinantes específicos ^17^ ^,^ ^18^ ^,^ ^19^. O objetivo deste trabalho é investigar um conjunto de fatores que influenciam o cumprimento da meta de compra de 30% da agricultura familiar no PNAE em duas regiões: Nordeste e Sul. 

A relevância do tema decorre do atual contexto socioeconômico, marcado pelo aumento da insegurança alimentar e pelas crises climáticas, que ameaçam a estabilidade da oferta de alimentos ^20^. Além disso, a aplicação de aprendizado de máquina permite análises mais precisas de grandes conjuntos de dados, ampliando o conhecimento sobre o PNAE e suas diferenças regionais.

## O cumprimento da “lei dos 30%” e as diferenças regionais

Um grande desafio da política pública está na implementação eficiente das regulamentações criadas de forma que os resultados esperados sejam alcançados com sucesso e de forma abrangente, ou seja, garantindo que toda a população foco da política seja contemplada.

Rodrigues et al. ^17^ exploraram a variação nos resultados do PNAE em função da capacidade institucional da gestão pública municipal. Outros trabalhos examinaram características do município, como o tamanho ^14^ ^,^ ^21^. Adicionalmente, pesquisas investigaram fatores relacionados aos agricultores, como a proximidade geográfica e a capacidade de atender aos requisitos das chamadas públicas, incluindo questões burocráticas, técnicas agrícolas, escala de produção e associativismo ^6^ ^,^ ^7^ ^,^ ^8^ ^,^ ^12^ ^,^ ^22^ ^,^ ^23^. 

Além disso, há autores que argumentam que as diferenças na implementação da política podem estar relacionadas às desigualdades regionais, fruto de heranças históricas que moldaram o uso da terra no país, bem como de fatores socioeconômicos e ambientais de cada região ^8^ ^,^ ^9^ ^,^ ^10^ ^,^ ^24^ ^,^ ^25^. Entretanto, são escassas análises que aprofundem a problemática das heterogeneidades territoriais, comparando o desempenho do PNAE entre diferentes regiões e considerando uma amostra abrangente de municípios.

No entanto, em um país de dimensões continentais como o Brasil, a implementação do PNAE necessariamente refletirá especificidades regionais. Sambuichi et al. ^26^ analisaram os perfis de produção familiar nas diferentes regiões e destacaram que, no Nordeste, a produção familiar é mais diversificada, mas apresenta baixo Valor Bruto da Produção (VBP), sendo grande parte dos produtos consumidos localmente, com pouco acesso ao mercado formal. Em contraste, a produção familiar na Região Sul é caracterizada por um alto VBP e uma diversidade produtiva mais orientada ao mercado, com investimentos direcionados ao aumento da produtividade e maior integração às cadeias de comercialização. 

Complementando essa análise, Souza et al. ^24^ observaram que, no Nordeste, a agricultura familiar é predominantemente voltada para o fornecimento de alimentos in natura ou minimamente processados, com pouca ou nenhuma participação de carnes. Essa produção é frequentemente associada à subsistência e à segurança alimentar das famílias produtoras. Já Machado et al. ^10^ identificaram que o cumprimento da meta do PNAE é particularmente elevado na Região Sul, onde cerca de 67% dos municípios adquiriram mais de 30% do valor repassado de produtos da agricultura familiar no período analisado.

Essas diferenças regionais oferecem subsídios para compreender os resultados observados a partir da implementação do PNAE e seus resultados nas distintas regiões do Brasil. Identificar e analisar os fatores que explicam o cumprimento ou não do programa em cada região é fundamental para identificar lacunas e propor melhorias que possam aumentar sua eficiência e eficácia. Nesse sentido, este estudo busca contribuir com uma análise aprofundada dessas variáveis nas regiões Sul e Nordeste, fornecendo bases para o aprimoramento da política nacional de alimentação escolar.

## Metodologia

Como apontado anteriormente, o objetivo deste trabalho é investigar os fatores que influenciam o cumprimento das normas estabelecidas nas diretrizes do PNAE e suas diferentes configurações nas regiões Sul e Nordeste. A escolha dessas regiões justifica-se: (i) pela relevância da agricultura familiar nas duas regiões, como *proxy* do impacto potencial do programa; e (ii) pela disparidade no cumprimento da meta, amplamente reconhecida pela literatura ^8^ ^,^ ^9^ ^,^ ^10^.

Para atingir o objetivo proposto, empregou-se uma metodologia baseada na revisão da literatura e na análise de dados secundários obtidos no site do FNDE ^27^, no Censo Agropecuário do Instituto Brasileiro de Geografia e Estatística (IBGE) ^28^, no Censo Escolar ^29^ e na *Pesquisa de Informações Básicas Municipais* ^30^. A base final é formada por dados de 2017, com exceção dos dados da última pesquisa citada.

Além das estatísticas descritivas, foi aplicado o método de árvores de classificação, uma técnica de aprendizado de máquina que identifica os fatores mais importantes para explicar o comportamento de determinada variável de interesse. A escolha dessa abordagem deve-se ao potencial do algoritmo para classificar os dados de forma supervisionada, com hipóteses mais flexíveis em relação aos métodos estatísticos tradicionais ^31^. Além de identificar os fatores determinantes, o método também permite ranqueá-los segundo sua importância relativa para a variável em análise. Essa abordagem possibilita uma análise mais aprofundada das singularidades regionais associadas à implementação do PNAE.

### Dados, variáveis e hipóteses

A base de dados do estudo compreende todos os 2.985 municípios das regiões Sul e Nordeste. O ano de referência principal é 2017, ano mais recente com informações disponíveis para caracterização da agricultura familiar, obtidas por meio do Censo Agropecuário. Apenas os dados obtidos na *Pesquisa de Informações Básicas Municipais* se referem a 2014, visto que as variáveis relativas à educação só estão disponíveis para esse ano. Após tratamento dos dados, que excluiu municípios com informações incompletas, a base final contou com 2.650 municípios. 

A variável dependente mede o sucesso do programa: o cumprimento da obrigatoriedade de que ao menos 30% dos recursos disponibilizados pelo FNDE sejam destinados à compra de produtos da agricultura familiar. Se esse valor é maior ou igual a 30%, então o município cumpre a norma e a variável dependente assume valor 1, caso contrário, ou seja, caso o valor seja menor do que o estabelecido pela lei, então a variável assume o valor 0.

As variáveis explicativas estão relacionadas aos diferentes fatores que podem influenciar o cumprimento da lei, classificadas em três dimensões: perfil demográfico, capacidades estatais e dados da agricultura familiar. O Quadro 1 descreve as variáveis e as hipóteses associadas a cada uma delas.

**Quadro 1:** Variáveis independentes: descrição e hipóteses.

DIMENSÃO	VARIÁVEL	DESCRIÇÃO	HIPÓTESE/JUSTIFICATIVA
Perfil demográfico	*região*	Conjunto de 5 variáveis binárias, uma para cada região, em que a observação assume valor 1 caso o município em questão pertença à região indicada e 0 caso contrário	A região em que o município se encontra é determinante para o percentual de compra realizado ^8^ ^,^ ^14^
	*dens_pop*	Definida pela razão entre a estimativa populacional e a estimativa da área territorial do município	A elevada densidade populacional pode implicar maiores dificuldades logísticas, associadas ao ordenamento urbanístico de cidades densas e à distância entre zonas urbanas e rurais ^8^ ^,^ ^14^ ^,^ ^15^
Capacidades estatais	*pib_percapita*	Razão entre o PIB municipal e a população do município	Municípios com elevado PIB *per capita* possuem maior capacidade financeira de complementação dos recursos transferidos pelo FNDE ^24^
	*rh_estatutario*	Número de servidores estatutários (considerando 2014 como referência). *Proxy* do tamanho do município e da capacidade técnico-administrativa para implementação do PNAE	Por um lado, o número de servidores estatutários associa-se ao tamanho do município. A literatura aponta que quanto maior o município, mais desafiadora a implementação do programa. Por outro lado, quanto maior o número de servidores públicos estatutários, maior a capacidade do município de implementar, monitorar e fiscalizar o cumprimento das diretrizes do PNAE ^25^
	*cae_2014*	Número de anos desde que o conselho foi criado (considerando 2014 como referência). *Proxy* da capacidade político-relacional do município para implementação do PNAE	Quanto maior o tempo transcorrido desde a criação do Conselho de Alimentação Escolar (CAE), maior a experiência do conselho e maior a sua capacidade de monitoramento e fiscalização do programa, aspecto fundamental para a eficácia das compras públicas ^14^ ^,^ ^26^
	*lei_educ_2014*	Variável binária que assume valor igual a 1 quando o município possui uma lei orgânica que define o percentual da receita destinado ao ensino público municipal e 0 caso contrário (considerando 2014 como referência)	A existência da lei orgânica indica o compromisso com a gestão educacional, já que reforça a responsabilidade municipal e a disponibilidade de recursos destinados ao ensino público e impede variações bruscas em caso de mudanças na gestão pública ^27^
	*org_gestor_2014*	Variável binária que assume valor igual a 1 quando há uma secretaria municipal de educação e 0 caso contrário (considerando 2014 como referência)	Quanto mais especializado o órgão responsável pela gestão do programa, maiores as capacidades disponíveis para implementação das políticas públicas educacionais ^28^
	*resp_recursos_2014*	Variável binária que assume valor igual a 1 quando há um órgão responsável pela gestão dos recursos da educação e 0 caso contrário (considerando 2014 como referência)	Um órgão especializado está mais atento às diretrizes do programa e comprometido com seu cumprimento, enquanto departamentos que lidam com todas as licitações municipais focam mais na eficiência de recursos e redução de custos. A existência de um órgão exclusivo para gestão dos recursos educacionais, portanto, aumenta a probabilidade de sua alocação adequada, conforme previsto em lei ^24^ ^,^ ^27^
Agricultura familiar	*af*	Proporção de estabelecimentos da agricultura familiar em relação ao total de estabelecimentos agropecuários no município	A presença da agricultura familiar no município não apenas indica a disponibilidade de alimentos produzidos pelo segmento, mas também a importância política relativa desse grupo ^29^
	*dap_prop*	Proporção de estabelecimentos da agricultura familiar que possuem DAP em relação ao total de estabelecimentos da agricultura familiar no município	Para oferta ao PNAE é preciso a DAP Logo, quanto maior a proporção de estabelecimentos da agricultura familiar que possuem DAP, maior a facilidade de obter alimentos no próprio município ^30^
	*cnpj_prop*	Proporção de estabelecimentos da agricultura familiar que possuem CNPJ em relação ao total de estabelecimentos da agricultura familiar no município	*Proxy* da formalização dos agricultores familiares, quanto maior a proporção de estabelecimentos da agricultura familiar com CNPJ, maior capacidade de acessar as chamadas públicas
	*valor_af*	Valor de produção da agricultura familiar em relação ao valor total de produção agropecuária do município	Um grande número de estabelecimentos da agricultura familiar não necessariamente implica elevada produção. Por isso, considera-se também o valor relativo da produção da agricultura familiar como indicativo da disponibilidade de alimentos produzidos pelo segmento e da importância política desse grupo ^25^
	*tec*	Proporção de estabelecimentos da agricultura familiar que recebem orientação técnica em relação ao total de estabelecimentos da agricultura familiar no município	A literatura aponta que entre as razões para que os municípios tenham dificuldade de comprar alimentos da agricultura familiar está a incerteza quanto à oferta de produtos na quantidade e qualidade requeridas. Logo, o acesso à orientação técnica é apontado como fator importante para mitigação das dificuldades ^9^ ^,^ ^25^ ^,^ ^31^ ^,^ ^32^ ^,^ ^33^. Espera-se que o acesso à orientação técnica esteja associado ao aumento da capacidade de gestão da propriedade, da produtividade agrícola e do acesso a políticas públicas ^34^ ^,^ ^35^
	*coop*	Proporção de estabelecimentos da agricultura familiar associados a cooperativa e/ou entidade de classe em relação ao total de estabelecimentos da agricultura familiar no município	Uma dificuldade comumente apontada pelos agricultores para a participação nas chamadas públicas são as exigências burocráticas ^23^. Nesse sentido, as cooperativas figuram como importantes instituições mediadoras da interlocução entre entidade executora e agricultores familiares ^33^ ^,^ ^36^. Ademais, o associativismo é essencial para aumentar a escala de produção, ganhar poder de barganha, agregar valor aos produtos finais e atingir mercados que individualmente seriam inacessíveis ^37^
	*fin*	Proporção de estabelecimentos da agricultura familiar que obtiveram financiamento em relação ao total de estabelecimentos da agricultura familiar	A agricultura familiar é um segmento tipicamente mais vulnerável a condições socioeconômicas e ambientais. Por esse motivo, há no Brasil programas que visam apoiar atividades desenvolvidas pela agricultura familiar, a partir da concessão de linhas de crédito adequadas às necessidades desse grupo. Espera-se que o acesso ao crédito esteja associado ao aumento da produtividade e à ampliação da escala na produção de alimentos, favorecendo a participação nas chamadas públicas ^38^
	*energia*	Proporção de estabelecimentos da agricultura familiar com acesso à energia em relação ao total de estabelecimentos da agricultura familiar no município	O acesso à energia elétrica está associado a dificuldades de produção e ao bem-estar social dos produtores mais vulneráveis ^16^. Espera-se, portanto, que quanto menor a proporção de produtores com acesso à energia, menor o grau de cumprimento da lei de 30% do PNAE
	*infra*	Variável categórica que assume valor de 0 a 4, sendo o valor 4 atribuído aos casos em que a maioria dos estabelecimentos da agricultura familiar do município fazem irrigação e possuem unidades armazenadoras e possuem tratores e possuem veículos	Pelos mesmos motivos apresentados nos casos das variáveis de orientação técnica, associação a cooperativas e financiamento, espera-se que estabelecimentos com melhor infraestrutura tenham maior capacidade de participar das chamadas públicas ^8^

CNPJ: Cadastro Nacional de Pessoa Jurídica; DAP: Declaração de Aptidão ao PRONAF; FNDE: Fundo Nacional de Desenvolvimento da Educação; PIB: produto interno bruto; PNAE: Programa Nacional de Alimentação Escolar.

Fonte: elaboração própria.

O estudo utilizou dados públicos secundários, sem qualquer informação que permita a identificação de indivíduos. Todas as bases utilizadas respeitam os princípios de transparência e ética no uso de dados públicos.

### Árvores de decisão e aprendizado de máquina

Segundo Varian ^32^, modelos lineares generalizados, como *logit* ou *probit*, são comumente utilizados para problemas de classificação binária. No entanto, devido à complexidade das variáveis do PNAE, este estudo optou por utilizar árvores de classificação, método que não pressupõe relações lineares, nem suposições explícitas sobre a distribuição dos dados. Além disso, o método lida melhor com interações entre variáveis, reduzindo o impacto da multicolinearidade ^33^.

O modelo organiza as observações em divisões sucessivas (“nós”), maximizando a separação das classes. A otimização ocorre pela minimização da impureza de Gini, que avalia a probabilidade de erro na classificação. A importância das variáveis é determinada por sua contribuição na redução dessa impureza. O desempenho do modelo é medido pela acurácia, intervalo de confiança e *no information rate* (NIR), sendo este o mínimo necessário para que o modelo seja considerado relevante.

A análise foi conduzida no software R (http://www.r-project.org), com o uso do pacote *caret* (Kuhn ^34^). A acurácia dos modelos foi estimada por meio de validação cruzada estratificada com 10 partições (*10-fold cross-validation*), o que permite avaliar a estabilidade e a capacidade de generalização do modelo. Para cada partição, foram calculadas as métricas de desempenho, sendo os resultados posteriormente agregados para obtenção de estimativas médias. Os intervalos de 95% de confiança (IC95%) da acurácia foram calculados com base na distribuição binomial exata (Clopper-Pearson), conforme implementado na função *confusionMatrix()* do mesmo pacote. Essa abordagem confere maior robustez à quantificação da incerteza associada às estimativas de acurácia, especialmente em contextos com distribuição desbalanceada.

## Resultados e discussão

Foram estimadas duas árvores de classificação com a mesma especificação de modelo, uma para a Região Sul e outra para a Região Nordeste. A separação por regiões foi motivada por resultados prévios de Fonseca et al. ^8^, nos quais o fator “região” emergiu como nó raiz da árvore, indicando sua relevância na diferenciação dos padrões de cumprimento da obrigatoriedade de alocação de 30% dos recursos do FNDE para a compra de produtos da agricultura familiar. 

Como explicitado na Tabela 1, 50% dos municípios brasileiros cumprem a meta. A Região Sul, no entanto, se destaca, com 77% dos municípios atendendo à exigência legal. Na Região Nordeste, onde há igualmente elevada representatividade da agricultura familiar, apenas 38% dos municípios destinam 30% ou mais dos repasses do FNDE à compra de produtos da agricultura familiar. 

**Tabela 1:** Estatísticas descritivas agregadas e por região. Brasil, 2017.

Variáveis	Brasil [n = 4,378]	Nordeste [n = 1,212]	Sul [n = 1,059]
Proporção média de compra da agricultura familiar	0,29 (0,21)	0,23 (0,16)	0,43 (0,24)
Municípios que cumprem o mínimo estabelecido	2,198 (50%)	464 (38%)	815 (77%)
População média por município	35,900 (211,039)	35,343 (118,782)	23,556 (67,486)
Média de alunos no município	4.667 (17.413)	6.199 (9.406)	2.545 (5.655)
*dens_pop*	77 (312)	80 (304)	68 (197)
*pib_percapita*	23.203 (20.912)	11.583 (11.984)	32.530 (18.083)
Número de estatutários	682 (2,221)	745 (1,205)	493 (931)
*lei_educ_2014* (0)	672 (15%)	205 (17%)	142 (13%)
*lei_educ_2014* (1)	3.706 (85%)	1.007 (83%)	917 (87%)
*org_gestor_2014* (0)	1.830 (42%)	431 (36%)	689 (65%)
*org_gestor_2014* (1)	2.548 (58%)	781 (64%)	370 (35%)
*resp_recursos_2014* (0)	2.191 (50%)	758 (63%)	368 (35%)
*resp_recursos_2014* (1)	2,187 (50%)	454 (37%)	691 (65%)
*cae_2014*	12,2 (5,6)	12,1 (5,6)	12,0 (5,7)
*dap_prop*	0,33 (0,21)	0,40 (0,21)	0,43 (0,19)
*cnpj_prop*	0,05 (0,13)	0,00 (0,00)	0,00 (0,01)
*af*	0,72 (0,14)	0,78 (0,10)	0,76 (0,13)
*valor_af*	0,40 (0,26)	0,53 (0,24)	0,47 (0,27)
*tec*	0,27 (0,23)	0,10 (0,10)	0,50 (0,22)
*energia*	0,86 (0,14)	0,79 (0,18)	0,90 (0,09)
*coop*	0,38 (0,22)	0,38 (0,20)	0,52 (0,22)
*fin*	0,17 (0,12)	0,14 (0,08)	0,29 (0,16)
*infra* (0)	3.503 (80%)	1.125 (93%)	695 (66%)
*infra* (1)	718 (16%)	87 (7,2%)	264 (25%)
*infra* (2)	146 (3,3%)	0 (0%)	94 (8,9%)
*infra* (3)	11 (0,3%)	0 (0%)	6 (0,6%)

Fonte: elaboração própria.

Nota: valores expressos como média (desvio padrão) ou frequência absoluta (percentual).

Variáveis: *lei_educ_2014* - existência, na Lei Orgânica Municipal, de dispositivo que define o percentual da receita destinado ao ensino público municipal; *org_gestor_2014* - existência de uma secretaria responsável exclusivamente pela educação; *resp_recursos_2014* - existência de um órgão responsável pela gestão dos recursos da educação; *cae_2014* - número de anos desde a criação do Conselho de Alimentação Escolar; *dap_prop* - proporção de estabelecimentos da agricultura familiar com Declaração de Aptidão ao PRONAF em relação ao total de estabelecimentos da agricultura familiar no município; *cnpj_prop* - proporção de estabelecimentos da agricultura familiar com CNPJ; *tec* - proporção de estabelecimentos da agricultura familiar que recebem assistência técnica; *energia* - proporção de estabelecimentos da agricultura familiar com acesso à energia elétrica; *coop* - proporção de estabelecimentos da agricultura familiar associados a cooperativas e/ou entidades de classe; *fin* - proporção de estabelecimentos da agricultura familiar que obtiveram financiamento; *infra* - variável categórica de 0 a 4, em que 4 representa municípios em que a maioria dos estabelecimentos da agricultura familiar realiza irrigação e possui unidades armazenadoras de tratores e veículos.

Tal disparidade em regiões onde o programa tem elevado potencial de impacto justifica a estimação de árvores regionais para uma análise mais refinada dos determinantes associados ao cumprimento da meta em contextos territoriais distintos. Os resultados são apresentados nas subseções a seguir.

### Região Nordeste

**Figura 1:**
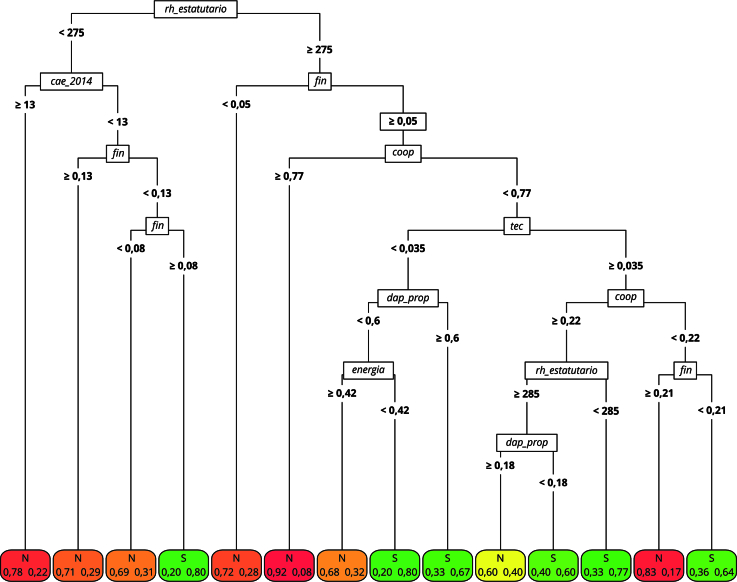
Árvore de classificação, cumprimento da medida. Região Nordeste, Brasil.

Ao avaliar os dados para a Região Nordeste, apresentados na Figura 1, nota-se que a primeira divisão da árvore se dá a partir da variável relativa ao número de servidores estatutários, que, apesar de medir a capacidade da gestão pública, controla o tamanho do município. Essa variável parece exercer grande influência na adesão à legislação do PNAE ^14^. Segundo a literatura, por um lado, municípios maiores enfrentam desafios adicionais relacionados à escala da demanda e às dificuldades logísticas. Por outro lado, em municípios menores, o principal desafio tende a ser a capacidade estatal para implementar a política de forma descentralizada ^8^ ^,^ ^10^ ^,^ ^18^ ^,^ ^21^.

Fonte: elaboração própria. Nota: variáveis: *rh_estatutario* - número de funcionários públicos estatutários; c*ae_2014* - número de anos desde a criação do Conselho de Alimentação Escolar; *fin* - proporção de estabelecimentos da agricultura familiar que obtiveram financiamento em relação ao total de estabelecimentos da agricultura familiar no município; *coop* - proporção de estabelecimentos da agricultura familiar associados a cooperativas e/ou entidades de classe; *tec* - proporção de estabelecimentos da agricultura familiar que recebem assistência técnica; *dap_prop* - proporção de estabelecimentos da agricultura familiar com Declaração de Aptidão ao PRONAF; *energia* - proporção de estabelecimentos da agricultura familiar com acesso à energia elétrica.

Logo, a hipótese associada a essa variável é ambígua. Por um lado, quanto maior o número de servidores, maior o município e maiores os desafios para o cumprimento da política. Por outro lado, um maior número de servidores estatutários pode implicar uma maior capacidade de implementar e monitorar o cumprimento das diretrizes do PNAE. Os resultados confirmam a importância dessa variável, tal como apontado por Machado et al. ^10^.

**Figura 2:**
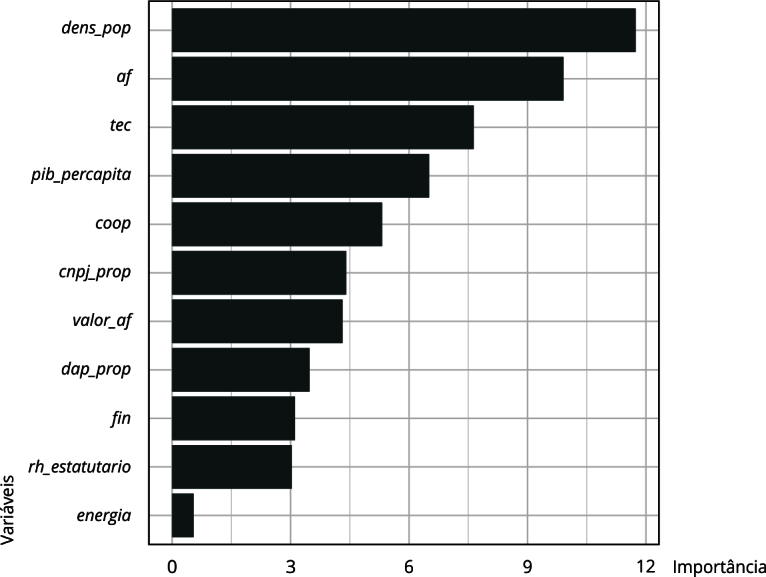
Importância das variáveis. Região Nordeste, Brasil.

A proporção de estabelecimentos com financiamento também é identificada como um divisor importante na árvore. Municípios com um elevado número de servidores estatutários e agricultores com reduzido acesso ao financiamento recebem uma classificação negativa, ou seja, municípios grandes e nos quais os produtores não possuem esse tipo de incentivo têm menos chances de cumprir o percentual previsto pela legislação, tal como discutido por Sambuichi et al. ^35^. Vale notar ainda que a variável financiamento foi identificada como a mais importante do modelo (Figura 2).

Fonte: elaboração própria. Nota: variáveis: *fin* - proporção de estabelecimentos da agricultura familiar que obtiveram financiamento em relação ao total de estabelecimentos da agricultura familiar no município; *rh_estatutario* - número de funcionários públicos estatutários; *dap_prop* - proporção de estabelecimentos da agricultura familiar com Declaração de Aptidão ao PRONAF; *coop* - proporção de estabelecimentos da agricultura familiar associados a cooperativas e/ou entidades de classe; *tec* - proporção de estabelecimentos da agricultura familiar que recebem assistência técnica; *energia* - proporção de estabelecimentos da agricultura familiar com acesso à energia elétrica; *cae_2014* - número de anos desde a criação do Conselho de Alimentação Escolar; *dens_pop* - estimativa da densidade populacional; *af* - proporção de estabelecimentos da agricultura familiar em relação ao total de estabelecimentos agropecuários; *valor_af* - proporção do valor de produção da agricultura familiar em relação ao valor total da produção agropecuária; *pib_percapita* - razão entre o PIB municipal e a população do município; *cnpj_prop* - proporção de estabelecimentos da agricultura familiar com CNPJ em relação ao total de estabelecimentos da agricultura familiar no município.

Além do número de funcionários públicos estatutários e da proporção de estabelecimentos que obtiveram financiamento, a proporção de estabelecimentos que possuem DAP (Declaração de Aptidão ao PRONAF - Programa Nacional de Fortalecimento da Agricultura Familiar) − substituída pelo Cadastro Nacional da Agricultura Familiar (CAF), em 2022 − e a proporção de estabelecimentos ligados a cooperativas e/ou entidades de classe também despontaram como fatores relevantes para explicar o cumprimento da lei da agricultura familiar no âmbito do PNAE. Essa análise, derivada da métrica de importância, sugere que, entre os municípios nos quais os produtores têm maior acesso ao financiamento, aqueles cuja proporção de estabelecimentos associados às cooperativas é maior ou igual a 77% são classificados negativamente. 

Esse resultado poderia ser considerado contraintuitivo, uma vez que o associativismo é geralmente considerado um fator com influência positiva sobre a participação em chamadas públicas ^6^ ^,^ ^7^ ^,^ ^10^ ^,^ ^12^ ^,^ ^14^ ^,^ ^36^. Uma hipótese plausível, no entanto, é que muitas cooperativas operam como entidades de grande escala, frequentemente voltadas para mercados externos ou para o fornecimento em nível nacional. No caso das cooperativas de agricultores familiares que participam do PNAE, sua atuação uniforme e abrangente pode reduzir a sensibilidade às variabilidades locais, limitando a eficácia dessa variável em capturar diferenças significativas no nível municipal ^8^. 

Os resultados também mostram que, em municípios onde a proporção de associativismo é inferior a 77%, a assistência técnica torna-se um fator crucial para o cumprimento da meta do PNAE, como já apontado por Souza-Esquerdo & Bergamasco ^12^ e Fonseca et al. ^8^. Sua relevância se deve ao reconhecimento da assistência técnica como aspecto fundamental para viabilizar a oferta de alimentos na quantidade e qualidade exigidas ^12^ ^,^ ^23^. Nos casos em que a proporção de produtores com acesso à assistência técnica é menor que 3,5%, a formalização dos produtores, medida pela proporção daqueles com DAP, passa a ser essencial para atingir a meta. Isso indica que, na ausência de cooperativas, é necessário garantir assistência técnica, e, na falta tanto de cooperativas quanto de assistência técnica, o cumprimento da legislação só será observado quando os produtores alcançarem um nível mais elevado de formalização. Em uma região onde a proporção média de produtores com DAP é de 40%, é necessário que esse percentual alcance pelo menos 60% para aumentar significativamente as chances de cumprimento da meta de compra de produtos da agricultura familiar.

O papel da variável que indica a proporção de estabelecimentos da agricultura familiar em relação ao total não é tão importante na análise dos municípios do Nordeste, tampouco o valor da produção desses agricultores. Essa hierarquia indica que importa menos a proporção de produtores familiares e mais a sua adequação aos critérios de participação no programa. Essa conclusão está em consonância com o perfil estabelecido para a região por Sambuichi et al. ^35^, especialmente no que diz respeito ao alto nível de produção para subsistência. Logo, se um município tem 90% de produtores familiares, mas nenhum, ou poucos, detêm a DAP, esta segunda condição limita a oferta e, portanto, o cumprimento da meta de compra.

O modelo para a Região Nordeste teve uma acurácia de 73% dentro do intervalo de confiança (entre 70,85% e 75,9%). Comparada com o NIR (61%), a acurácia atende o filtro de relevância.

### Região Sul

**Figura 3:**
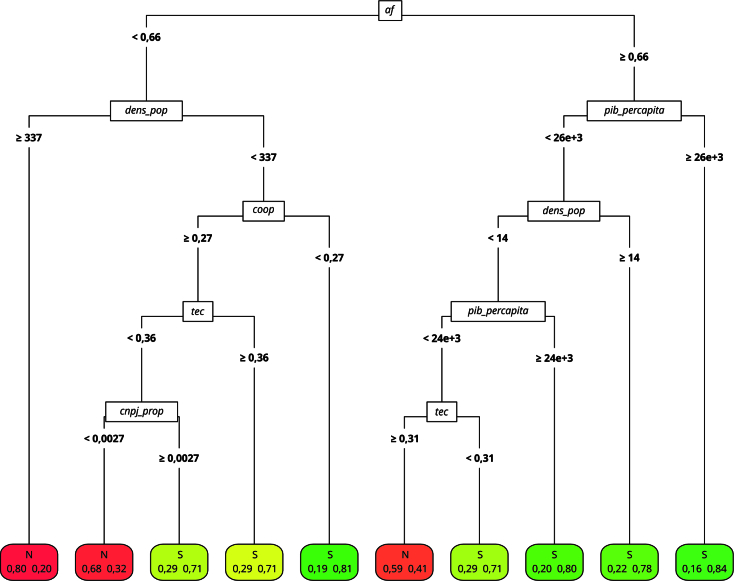
Árvore de classificação, cumprimento da medida. Região Sul, Brasil.

A árvore de classificação gerada com os dados da Região Sul (Figura 3) se diferencia notavelmente da obtida a partir de dados do Nordeste. No nó raiz, destaca-se a variável referente à proporção de estabelecimentos da agricultura familiar sobre o total de estabelecimentos agrícolas do município, indicando a disponibilidade de alimentos para aquisição pelo programa. Municípios onde essa proporção é inferior a 66% apresentam maior probabilidade de descumprir a regra, enquanto aqueles com maior participação da agricultura familiar tendem a cumprir a meta.

Fonte: elaboração própria. Nota: variáveis: *af* - proporção de estabelecimentos da agricultura familiar em relação ao total de estabelecimentos agropecuários; *dens_pop* - estimativa da densidade populacional; *pib_percapita* - razão entre o PIB municipal e a população do município; *coop* - proporção de estabelecimentos da agricultura familiar associados a cooperativas e/ou entidades de classe em relação ao total de estabelecimentos da agricultura familiar no município; *tec* - proporção de estabelecimentos da agricultura familiar que recebem assistência técnica; *cnpj_prop* - proporção de estabelecimentos da agricultura familiar com CNPJ.

A densidade populacional também se mostrou crucial, tendo sido identificada como a variável mais relevante para classificar os municípios. Em municípios com baixa presença da agricultura familiar, uma densidade superior a 337 habitantes/km^2^ está fortemente associada ao não cumprimento da meta. Já em municípios menos densos, há maior variabilidade na decisão final, mas a probabilidade de adesão ao programa aumenta, dado o número de nós finais classificados positivamente. Assim, municípios mais densos e com poucos agricultores familiares apresentam menores chances de cumprir a meta de compra, tal como discutido por Fonseca et al. ^8^.

Outro fator determinante é a proporção de estabelecimentos da agricultura familiar que recebem assistência técnica, evidenciando a atuação do Estado para fomentar a participação do setor no programa. No Sul, essa variável superou a relevância do acesso ao financiamento, o que reforça a assistência técnica como instrumento estratégico para a inclusão de produtores familiares em mercados institucionais, como o PNAE.

A maturidade da agricultura familiar na região, conforme destacado por Sambuichi et al. ^26^, também explica esse resultado. A elevada proporção de estabelecimentos com DAP (43%) sugere que os agricultores necessitam menos de financiamento e mais de suporte técnico. Machado et al. ^10^ apontam que a escassez de profissionais no quadro técnico e o desconhecimento da legislação impactam a adesão à *Lei nº 11.947/2009* ^4^ no Rio Grande do Sul, alinhando-se aos achados deste estudo.

Apesar das diferenças contextuais, a assistência técnica figura como um fator comum e determinante para o cumprimento da legislação do PNAE em ambas as regiões. Mesmo com níveis distintos de cobertura, esse fator mostrou-se um elemento importante para viabilizar a participação da agricultura familiar nas compras públicas, evidenciando seu papel estratégico no cumprimento da meta de aquisição mínima no âmbito do PNAE. No entanto, a proporção de estabelecimentos da agricultura familiar assistidos é significativamente maior no Sul em comparação ao Nordeste. Essa desigualdade pode influenciar diretamente a capacidade dos agricultores de se organizarem, se qualificarem e atenderem aos critérios das chamadas públicas, influenciando diretamente o sucesso na implementação do programa. 

**Figura 4:**
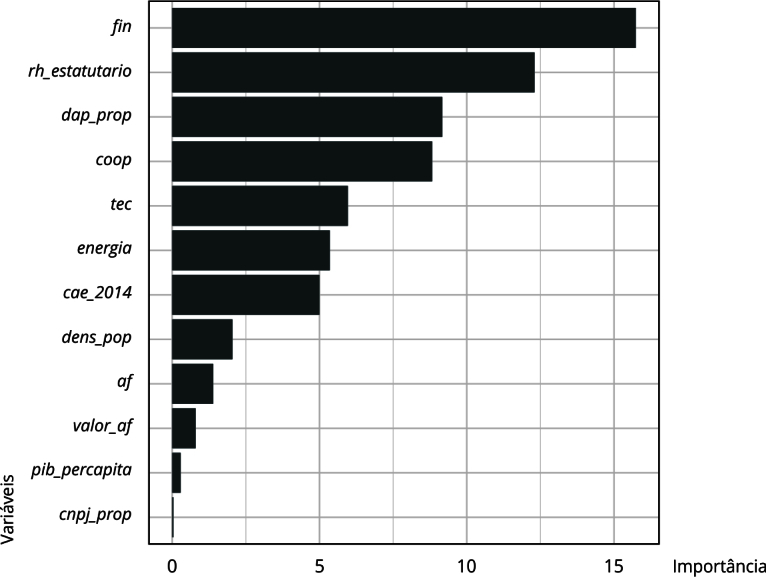
Importância das variáveis. Região Sul, Brasil.

Ademais, no que diz respeito à importância das variáveis (Figura 4), diferentemente do Nordeste, o PIB *per capita* municipal emergiu como um fator relevante no Sul, especialmente em municípios com baixa densidade populacional. Observou-se que, quanto maior o PIB *per capita*, maiores as chances de cumprimento da meta de compra, indicando que a capacidade financeira dos municípios pode ser decisiva para superar os custos de transação e viabilizar a implementação da política, mesmo em localidades com menor economia de escala.

Fonte: elaboração própria. Nota: variáveis: *dens_pop* - estimativa da densidade populacional; *af* - proporção de estabelecimentos da agricultura familiar em relação ao total de estabelecimentos agropecuários; *tec* - proporção de estabelecimentos da agricultura familiar que recebem assistência técnica em relação ao total de estabelecimentos da agricultura familiar no município; *pib_percapita* - razão entre o PIB municipal e a população do município; *coop* - proporção de estabelecimentos da agricultura familiar associados a cooperativas e/ou entidades de classe; *cnpj_prop* - proporção de estabelecimentos da agricultura familiar com CNPJ; *valor_af* - proporção do valor de produção da agricultura familiar em relação ao valor total da produção agropecuária; *dap_prop* - proporção de estabelecimentos da agricultura familiar com Declaração de Aptidão ao PRONAF em relação ao total de estabelecimentos da agricultura familiar no município; *fin* - proporção de estabelecimentos da agricultura familiar que obtiveram financiamento; *rh_estatutario* - número de funcionários públicos estatutários; *energia* - proporção de estabelecimentos da agricultura familiar com acesso à energia elétrica em relação ao total de estabelecimentos da agricultura familiar no município.

Quanto ao desempenho do modelo para a região, foi alcançada uma acurácia de 83,76%, dentro do IC95% (81,4% a 85,93%), explicada pela homogeneidade dos dados, conforme indicado pelo NIR (76%). A árvore não apresentou indícios de *overfitting*, embora aprimoramentos no modelo possam ser explorados em pesquisas futuras.

## Conclusão

Este trabalho analisou os fatores determinantes para o cumprimento da meta de destinar 30% dos recursos federais transferidos pelo FNDE aos municípios, no âmbito do PNAE, para a compra de alimentos da agricultura familiar, com foco nas diferenças entre as regiões Sul e Nordeste.

Utilizando aprendizado de máquina, especificamente árvores de classificação, os resultados confirmaram a influência de fatores históricos e regionais no cumprimento das diretrizes do PNAE, corroborando a literatura existente. A análise evidenciou diferenças significativas entre as regiões, não apenas em termos de árvores de decisão, mas também na relevância atribuída às variáveis com influência sobre a política.

No Nordeste, o cumprimento está mais ligado a fatores estruturais do desenvolvimento agrícola, como a proporção de estabelecimentos da agricultura familiar com acesso a financiamento e com a DAP. Esses achados indicam um estágio mais incipiente de implementação da política, em que os agricultores ainda dependem de apoio organizacional e de recursos financeiros para ampliar sua participação no PNAE. Já no Sul, a adesão se relaciona a características municipais, como densidade populacional, e à capacidade estatal, representada pelo PIB *per capita*. A assistência técnica e o associativismo se destacam em ambas regiões, evidenciando sua relevância para viabilizar o acesso às chamadas públicas do PNAE, ainda que haja um perfil mais autônomo dos agricultores e um estágio mais avançado na implementação da meta de compra da agricultura familiar no Sul.

Conclui-se que fatores históricos relacionados à estrutura histórica de ocupação e uso da terra, combinados com as particularidades da agricultura familiar e da capacidade estatal, explicam as diferenças entre as duas regiões. Esses achados reforçam a importância de políticas públicas flexíveis e adaptáveis que considerem especificidades regionais para uma implementação mais equitativa e eficaz do programa. Uma abordagem homogênea pode gerar resultados desiguais, reforçando a necessidade de estratégias ajustadas às necessidades locais.

Por fim, embora o estudo traga contribuições relevantes, há limitações, como a dependência de dados agregados e a escolha de variáveis que podem não capturar toda a complexidade do PNAE. A partir de entrevistas com gestores públicos, pesquisas futuras podem integrar dados qualitativos e aprofundar a compreensão sobre os desafios e oportunidades na implementação da política a nível local, subsidiando decisões mais alinhadas às realidades intrarregionais.

## Data Availability

Os dados de pesquisa estão disponíveis mediante solicitação ao autor de correspondência.
